# 49, XXXYY: Parental Origin, Occurrence, and Clinical Phenotypes

**DOI:** 10.1155/genr/1368153

**Published:** 2025-07-21

**Authors:** Yufang Du, Liangrong Liao, Xianda Wei, Yunting Ma, Meizhen Shi, Chunyan Li, Juliang Liu, Wenting Lin, Hao Zeng, Shaoke Chen, Baoheng Gui

**Affiliations:** ^1^Center for Medical Genetics and Genomics, The Second Affiliated Hospital of Guangxi Medical University, Nanning, Guangxi, China; ^2^The Guangxi Health Commission Key Laboratory of Medical Genetics and Genomics, The Second Affiliated Hospital of Guangxi Medical University, Nanning, Guangxi, China; ^3^Division of Neonatology, Department of Pediatrics, Guigang City People's Hospital, Guigang, Guangxi, China; ^4^The Second School of Medicine, Guangxi Medical University, Nanning, Guangxi, China; ^5^Department of Clinical Laboratory, Guigang City People's Hospital, Guigang, Guangxi, China; ^6^Department of Pediatrics, The Second Affiliated Hospital of Guangxi Medical University, Nanning, Guangxi, China

**Keywords:** copy number variation, parental origin, pentasomy, sex chromosome, short tandem repeats

## Abstract

49, XXXYY is a rare form of sex chromosomal aneuploidy that has been reported in 11 cases worldwide. The parental origin of the extra sex chromosomes and the specific clinical features of this condition remain unclear. We recruited a case with 49, XXXYY and performed genome-wide copy number variation analysis using next-generation sequencing. In addition, the parental origin of the extra sex chromosomes was determined through short tandem repeats (STRs) locus genotyping. Furthermore, a comprehensive review and comparison of clinical phenotypes were conducted among 12 cases with 49, XXXYY. The patient exhibited a karyotype of 49, XXXYY without any mosaic patterns. No pathogenic microdeletions or microduplications (> 100 kb) were identified in autosomes 1–22. Analysis of the STR loci revealed that two of three X chromosomes originated from father. This suggests that the nondisjunction of chromosomes X and Y during stages I and II of meiotic spermatogenesis led to the production of an abnormal sperm with XXYY. Subsequently, fertilization of a normal oocyte with this abnormal sperm resulted in an abnormal zygote with pentasomy XXXYY. The main clinical features observed in these cases included varying degrees of mental retardation, minor facial dysmorphology, and gonadal or endocrine abnormalities. In conclusion, 49, XXXYY is a rare chromosomal disorder characterized by mental retardation and facial dysmorphology. Nondisjunction of chromosomes X and Y during stages I and II of meiotic spermatogenesis is a critical factor contributing to the development of this abnormal karyotype.

## 1. Introduction

Sex chromosome aneuploidy is a prevalent chromosomal disorder characterized by the addition or deletion of one or more sex chromosomes. Four main causes of chromosomal disorders are chemical factors (such as drugs, pesticides, industrial poisons, and food additives), physical factors (such as high-level radiation exposure), biological factors (including biotoxoids and viruses), and maternal age [1]. Sex chromosome aneuploidy encompasses monosomy, trisomy, tetrasomy, and pentasomy. Monosomy (45, X) and trisomy (47, XXX, 47, XXY, and 47, XYY) are well documented due to their relatively high incidence of 1 in 400 newborns [2]. However, there is a lack of knowledge regarding sex chromosomal tetrasomies, such as 48, XXXX, 48, XXXY, 48, XXYY, and 48, XYYY, and pentasomies, such as 49, XXXXX, 49, XXXXY, 49, XXXYY, 49, XXYYY, and 49, XYYYY, due to their rarity. Cases with more than 50 chromosomes or more than six sex chromosomes have not been reported thus far.

49, XXXYY is one rare form of sex chromosomal pentasomy. To date, only 11 cases of 49, XXXYY have been reported worldwide, exhibiting varied phenotypes, including normal to tall stature, mild to severe intellectual disability, minor facial or truncal deformations, reproductive system dysplasia, and endocrine problems [3–13]. However, the pathogenesis of 49, XXXYY and the parental origin of the extra sex chromosomes remain unclear.

This study aims to describe the clinical manifestations of a 51-day-old boy diagnosed with 49, XXXYY, to determine the copy number variation (CNV) of the entire genome using high-throughput sequencing, to identify the parental origin of the five sex chromosomes (XXXYY) and three autosomes (13, 18, and 21) using short tandem repeats (STRs) locus genotyping, and to provide a brief review of previously reported cases of 49, XXXYY from various regions around the world.

## 2. Materials and Methods

### 2.1. Medical Records of the Present Case

The patient, immediately after birth, was transferred to the Neonatal Department of the Maternal and Children Hospital of Guigang (Guigang, China) within 43 min due to low birth weight and poor feeding appearance. He received supportive care at the facility for 7 days. On the 50th day, the parents brought him to the Endocrinic Genetic Metabolic Department of the Second Affiliated Hospital of Guangxi Medical University (Nanning, China) for further consultation and therapy. The patient was the first child of a healthy, nonconsanguineous Chinese couple, with his mother being 35 years old and his father being 33 years old at the time of his birth. The family had no notable medical history, and the pregnancy progressed without any complications, culminating in a 38th-week spontaneous vaginal delivery.

All protocols were approved by the Ethical Review Committee of the Second Affiliated Hospital of Guangxi Medical University (Nanning, China) (Approval number 2021-KY-0369). Written informed consent was obtained from all subjects in accordance with the Declaration of Helsinki.

### 2.2. Methods

The karyotype was determined by two experienced analysts after examining a total of 40 metaphase cells for each case, utilizing the MetaSight G120 and AutoVision software (DIAGENS, Hangzhou, China). CNV sequencing was conducted on the NextSeq CN500 platform, utilizing the NextSeq CN500 High-throughput sequencing kit (BerryGenomics, Hangzhou, China). STR genotyping was performed using capillary electrophoresis analysis, and the data were analyzed using the 3500 xL genetic analyzer (Life Technologies, California, USA).

## 3. Results

### 3.1. Clinical Evaluation and Test Results of the Patient

The birth weight of the infant was 2480 g, and the length was 47 cm. No external genital deformities were observed although there was swelling in the scrotal area. The newborn received Apgar scores of 10 at 1, 5, and 10 min after birth. Initial diagnostic tests, including x-ray, echocardiogram, and brain MRI, yielded negative results within the first week. An ultrasound of the cranial cavity revealed the presence of two mixed echo masses in the bilateral thalamus caudate incisura, with the left mass measuring 18 × 8 mm and the right mass measuring 19 × 8 mm. Karyotyping analysis of peripheral blood lymphocytes confirmed an abnormal karyotype of 49, XXXYY.

Physical examinations conducted on the 51st day revealed normal neurodevelopment, with the infant weighing 3400 g and measuring 38 cm in crown-rump length. Apart from the notable feature of posteriorly rotated ears (Supporting Figure 1), no other facial dysmorphia was observed. Hormone tests indicated lower estradiol levels and higher levels of prolactin, progesterone, and testosterone. The color ultrasound examination revealed that the size of the right testicle was 1.3 × 0.8 × 0.7 cm, while the left testicle measured 1.2 × 1.0 × 0.7 cm. In addition, the echocardiogram showed evidence of atrial-level shunting from left to right although a definitive conclusion had not been reached.

### 3.2. Results of Karyotyping and CNV Sequencing

**Figure 1:**
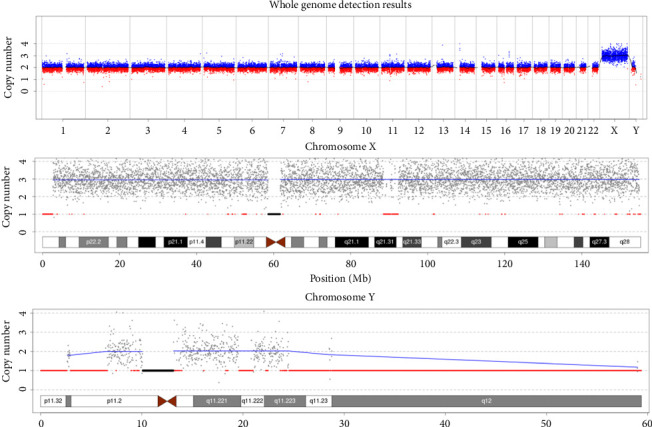
Results of CNV sequencing of the patient.

The patient's karyotype was determined to be 49, XXXYY, with no observed mosaicism (Supporting Figure 2). Sequencing analysis of the patient's autosomes 1–22 did not reveal any known pathogenic CNVs (> 100 kb). The patient exhibited a copy number of 3 for the X chromosome and 2 for the Y chromosome, indicating a triple X and double Y dosage compared with the normal male level (Figure 1). Both of the patient's parents had normal karyotypes.

### 3.3. Results of STR Genotyping

**Figure 2:**
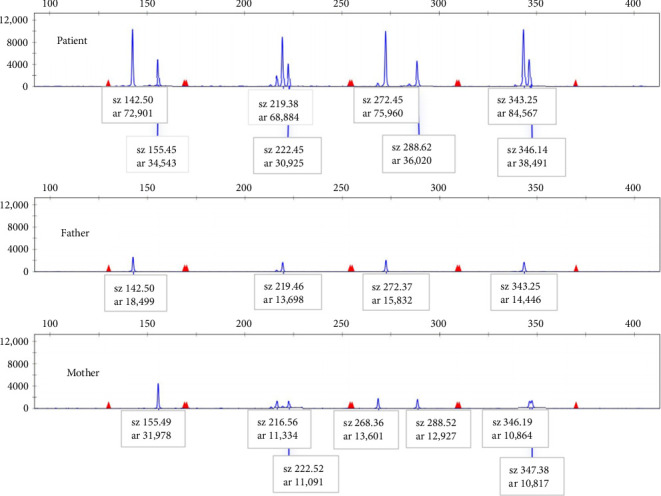
Capillary electrophoresis results of four X-STR locus.

**Figure 3:**
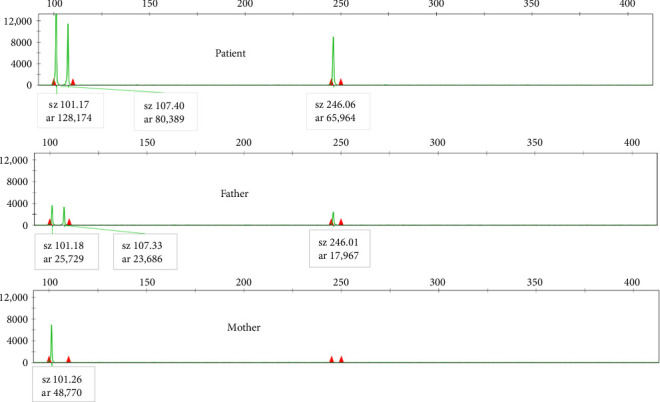
Capillary electrophoresis results of three alleles in AMXY and SRY genes.

The sizes of the products for all selected STR loci are listed in Supporting Table 1. The abundance of each STR locus product was determined by capillary electrophoresis peak area. As depicted in Figure 2, the abundance of X-STR locus products from the paternal origin was approximately double that of the maternal origin, indicating two paternal X chromosomes and one maternal X chromosome. In Figure 3, the abundance of the X-related locus product (sz101) was 128,174, while the abundance of the Y-related locus product (sz107) was 80,389, resulting in a ratio of approximately 3:2 (128,174/80,389), suggesting a numerical ratio of 3:2 between X and Y, which concurred with the karyotype. The haploid typing-linkage analysis of the family is presented in Supporting Figure 3.

### 3.4. Summary of Phenotypes of Twelve 49, XXXYY Cases

There have been 11 previously reported cases of 49, XXXYY, including one fetus, five children, and five adults, exhibiting varying phenotypes. Among these cases, all except one 3.5-year-old patient with ambiguous external genitalia were phenotypically male. The 10 postnatal cases, ranging in age from 12 days old to 42 years old, displayed a range of normal to tall stature and experienced mild to severe mental retardation. In addition, eight cases exhibited delays in motor or speech development, two cases demonstrated low frustration tolerance, and one case was diagnosed with autism. Other phenotypic characteristics included (1) gonadal or endocrine problems, such as hypogenitalism, small penis, cryptorchidism, and gynecomastia; (2) facial dysmorphology, including a protruding lower jaw, micrognathia, narrow palpebral fissures, prominent forehead, prominent supraorbital ridges, short upper lip, deeply set eyes, broad nasal bridge, epicanthic folds, and posteriorly rotated ears; and (3) other symptoms or conditions such as congestive heart failure, mild acromegaly, diabetes, asthma, hypothyroidism, and depression. In the present study, the case we report showed no notable facial malformation except for the posteriorly rotated ears on his 51th day. However, when he was 2.5 years old, the patient can speak only several simple words like “papa” and “mama” and walk dependently. No apparent mental anomaly was observed in the latest telephone follow-up visit.

## 4. Discussion

Sex chromosomal aneuploidy affects individuals of all races, with males and females equally affected. The occurrence of 49, XXXYY, a relatively rare sex chromosome numerical aberration, has been documented globally. To the best of our knowledge, the case we present here is the twelfth reported 49, XXXYY case worldwide and the second documented case in China, characterized by the absence of obvious dysmorphology. Among the previously reported cases of 49, XXXYY (age > 12 days), eight individuals exhibited varying degrees of mental retardation. In our case, the patient's age precludes drawing conclusions about his mental condition, necessitating long-term follow-up. The parental age of the all reported cases of 49, XXXYY ranged from 20 to 44 (Table 1). It appears that the occurrence of 49, XXXYY is unrelated to parental age. Nevertheless, further studies are needed to explore the relationship between parental age and the incidence of sex chromosome pentasomy.

### 4.1. Sex Chromosome Dosage and X Chromosome Inactivation

The severity of mental retardation in sex chromosome aneuploidy individuals is believed to be closely correlated with the number of additional X chromosomes [14]. Individuals with X chromosome pentasomy generally exhibit more severe cognitive symptoms compared with those with tetrasomy [15]. Y polysomy exhibits variable phenotypes, mental retardation, skeletal malformation, and azoospermia are three common clinical symptoms in Y polysomy individuals [16]. However, the clinical impact of Y chromosome dosage is still unclear due to the rarity of Y polysomy cases.

Autosomal aneuploidies are typically lethal, except for trisomy 21, while sex chromosomal aneuploidies always survive. It was proposed that one out of every two somatic X chromosomes inactivated randomly early in embryonic development, while a small number of genes can evade inactivation and be expressed, leading to dosage imbalance and phenotypic differences. The severity of phenotypic abnormalities is directly proportional to the number of excess X chromosomes present [17, 18].

### 4.2. Chromosomal Nondisjunction and Aneuploidy

The mechanisms underlying chromosomal nondisjunction are complex. Defects in the attachment of spindle microtubules to kinetochores, such as the presence of extra centrosomes, hyperstable kinetochore-microtubule attachments, and reduced centromere cohesion, can contribute to chromosomal nondisjunction [19]. Moreover, recent research by Klaassen et al. suggested that the frequency of chromosomal segregation errors is influenced by the nuclear chromosomal location. Specifically, peripheral chromosomes are more prone to incorrect segregation due to the longer time required for congression compared with central chromosomes. The study found that chromosomes 1–5, 8, 11, and X in tumor cells exhibited significantly higher frequencies of segregation errors compared with chromosomes 14, 15, and 19–22 [20]. Nondisjunction events during meiosis can lead to chromosomal gains or losses in oocytes or sperms, thus increasing the likelihood of aneuploidy upon fertilization [21].

### 4.3. Parental Origin of Additional Sex Chromosome

Differences in the contribution of paternal and maternal chromosomes to aneuploidy can be attributed to variations in the initiation, duration, and outcome of sex-specific meiotic processes [22]. One previous study has reported that nearly 50% of the cases with 47, XXY (Klinefelter syndrome) have a paternal origin of the extra X chromosome, while in other trisomies, such as Trisomy 21 (Down syndrome) and X trisomy, maternal origin accounts for 90%–95% of the additional chromosomes [23]. The situation in sex chromosomal tetrasomy appears to be more diverse. For instance, in five cases of 48, XXXX, the extra X chromosomes were all of maternal origin. Moreover, in two of these cases, uniparental origin was identified, indicating the absence of paternal sex chromosomes [24–26]. In addition, in eight cases of 48, XXYY, both the extra X and Y chromosomes were contributed by the father [8, 25]. In the case of 48, XXXY, two additional X chromosomes could originate from either the father or the mother [8, 25]. In six cases of 49, XXXXX and twenty-one cases of 49, XXXXY, the additional X chromosomes were identified as maternal origin [8, 24–29]. However, there are no reported studies investigating the origin of the extra sex chromosomes in 49, XXXYY, 49, XXYYY, and 49, XYYYY.

**Figure 4:**
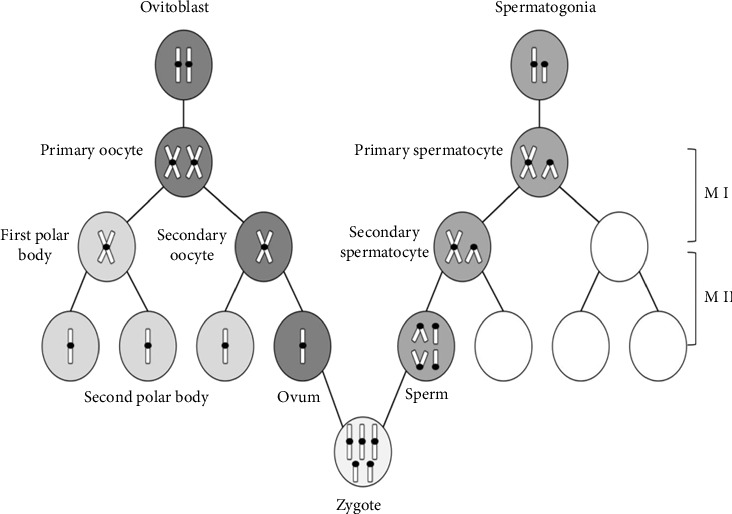
A deduced generation mechanism of zygote with XXXYY.

This study is the first to investigate the parental origin of sex chromosomes in individual with 49, XXXYY. The STR data indicated that the presence of two extra X chromosomes and two Y chromosomes was of paternal origin. There were mis-segregation events observed in both the X and Y chromosomes in the sperm although the exact mechanism remains unclear. We propose a possible generation process for sex chromosome pentasomy (XXXYY): during meiosis I, the X and Y chromosomes, which contain duplicated genetic material, fail to segregate into two daughter cells. Then, during meiosis II, the segregation of the X and Y chromosomes stops in the anaphase, resulting in sperm with four sex chromosomes (XXYY). When this abnormal sperm fertilizes a normal ovum carrying one X chromosome, a zygote with XXXYY is formed (Figure 4).

In conclusion, the phenotype of the 49, XXXYY individuals examined in this study was deemed largely normal, with the exception of uncertain mental conditions. It is noteworthy that, for the first time, we have established that the extra X chromosomes and two Y chromosomes in 49, XXXYY are of paternal origin. Further long-term follow-up and acquisition of more detailed information are essential to gain a deeper understanding of the effects and implications of additional sex chromosomes.

## Figures and Tables

**Table 1:** Clinical phenotypes of 49, XXXYY.

	Case 1	Case 2	Case 3	Case 4	Case 5	Case 6	Case 7	Case 8	Case 9	Case 10	Case 11	The current case
Reported year	1963	1974	1981	1982	1986	1995	2013	2019	2022	2023	2024	2025
Phenotypic gender	Male	Male-like	Male	Male	Male	Male	Male	Male	Male	Male	Male	Male
Age (year)	26	3.5	29	23 weeks	42	6	3.5	12 days	25	1.5	29	51 days
Height (cm)	192	Normal	176	29	170	113	/	/	182.5	73	180.8	53
Mental retarded^†^	+	+	++	/	+	+	+	/	+	/	+	/
Motor development delay^†^	+	+	/	/	+	+	+	/	+	+	+	/
Speech/language development delay^†^	/	+	/	/	+	++	/	/	+	+	+	/
Head/facial dysplasia^†^	+	+	+	+	+	+	/	/	+	+	+	+
Truncal/limbs dysplasia^†^	+	/	+	/	+	+	/	/	+	+	+	/
Urinary/reproductive system dysplasia^‡^	1, 2	3	1, 4	/	4	1, 2	4	4	1	1, 2, 4	1, 4	/
Other symptoms/diseases^‡^	5, 6	/	7	/	8, 9	9, 10	/	/	11, 12	/	13, 14	/
Maternal age	20	30	38	44	41	30	< 30	32	31	25	21	35
Paternal age	/	35	44	35	/	32	< 30	30	32	28	21	33

^†^“+”: symptomatic positive, “/”: not mentioned.

^‡^(1) Small penis, (2) small testes, (3) ambiguous external genitalia, (4) cryptorchidism, (5) congestive failure, (6) acromegaly, (7) diabetes, (8) depression, (9) low frustration tolerance, (10) asthma, (11) autism, (12) hypothyroidism, (13) primary hypogonadism, (14) essential tremor.

## Data Availability

Research data are not shared.

## Supporting Information

Supporting Information 1 Ethics approval notice (2021-KY-0369).

Supporting Information 2 Supporting Figure 1. Photograph of the 51-day-old boy with 49, XXXYY.

Supporting Information 3 Supporting Figure 2. Karyotype of the patient (49, XXXYY).

Supporting Information 4 Supporting Figure 3. Haploid typing-linkage analysis.

Supporting Information 5 Supporting Table 1. Results of STR.
