# Supplementary material for: 49, XXXYY: Parental Origin, Occurrence, and Clinical Phenotypes
Source: Genet Res (Camb). 2025 Jul 21;2025:1368153. doi: 10.1155/genr/1368153 (PMC12316498; doi:10.1155/genr/1368153)
Supplement: Supporting Information 1 — Ethics approval notice (2021-KY-0369). [file 1368153.f1.pdf]

THE SECOND AFFILIATED HOSPITAL of GUANGXI MEDICAL UNIVERSITY
ETHICAL REVIEW COMMITTEE
Approval Notice

Approval Number: 2021-KY(0369)

Title: 49,XXXYY: parental origin, occurrence, and clinical phenotypes

Research Contents: We recruited a case with 49,XXXYY and performed genome-wide copy number variation analysis using next-generation sequencing. Additionally, the parental origin of the extra sex chromosomes was determined through short tandem repeats (STR) locus genotyping. Furthermore, a comprehensive review and comparison of clinical phenotypes were conducted among eight cases with 49,XXXYY.The patient exhibited a karyotype of 49,XXXYY without any mosaic patterns. No pathogenic micro-deletions or micro-duplications (>100kb) were identified in autosomes 1-22. Analysis of the STR loci revealed that two of the three X chromosomes originated from the father. This suggests that the non-disjunction of chromosomes X and Y during stages I and II of meiotic spermatogenesis led to the production of an abnormal sperm with XXYY. Subsequently, fertilization of a normal oocyte with this abnormal sperm resulted in an abnormal zygote with pentasomy XXXYY. The main clinical features observed in these cases included varying degrees of mental retardation, minor facial dysmorphology, and gonadal or endocrine abnormalities.

Applicant: Baoheng Gui, Yufang Du.

Application Department: Center for Medical Genetics and Genomics， The Second Affiliated Hospital of Guangxi Medical University

Acknowledgement: This work was supported in part by the National Natural Science Foundation of China (82001531 and 81860272 to BG), Guangxi Major Research Programme (AB22035013 to BG), Guangxi Natural Science Foundation (2018GXNSFAA281067 to BG), Initial Scientific Research Fund for Advanced Talents from The Second Affiliated Hospital of Guangxi Medical University (2019112 to BG), and Special Scientific Research Fund of Guangxi Ten-Hundred-Thousand Talents Project (2021186 to BG).

Date of Application: 2021.5.6

Date of Approval: 2021.5.10

Conclusion: This project fully considered and protected the rights and interests of the study objects. It meets the criteria of Ethical Review Committee. The Medical Ethics Committee of Second Affiliated Hospital of Guangxi Medical University has approved the protocol.

Signature:
Jianfeng Zhang
Director of Ethical Review Committee
The Second Affiliated Hospital of
Guangxi Medical University
Date: 2021.5.10

NO.166, Daxuedong Road, Nanning, Guangxi, P.R.China, 530007
Tel: +86 0771 3277533 Fax: +86 0771 3277285 E-mail: ykdefykyb@163.com
