# Supplementary figures and images for: 49, XXXYY: Parental Origin, Occurrence, and Clinical Phenotypes
Source: Genet Res (Camb). 2025 Jul 21;2025:1368153. doi: 10.1155/genr/1368153 (PMC12316498; doi:10.1155/genr/1368153)

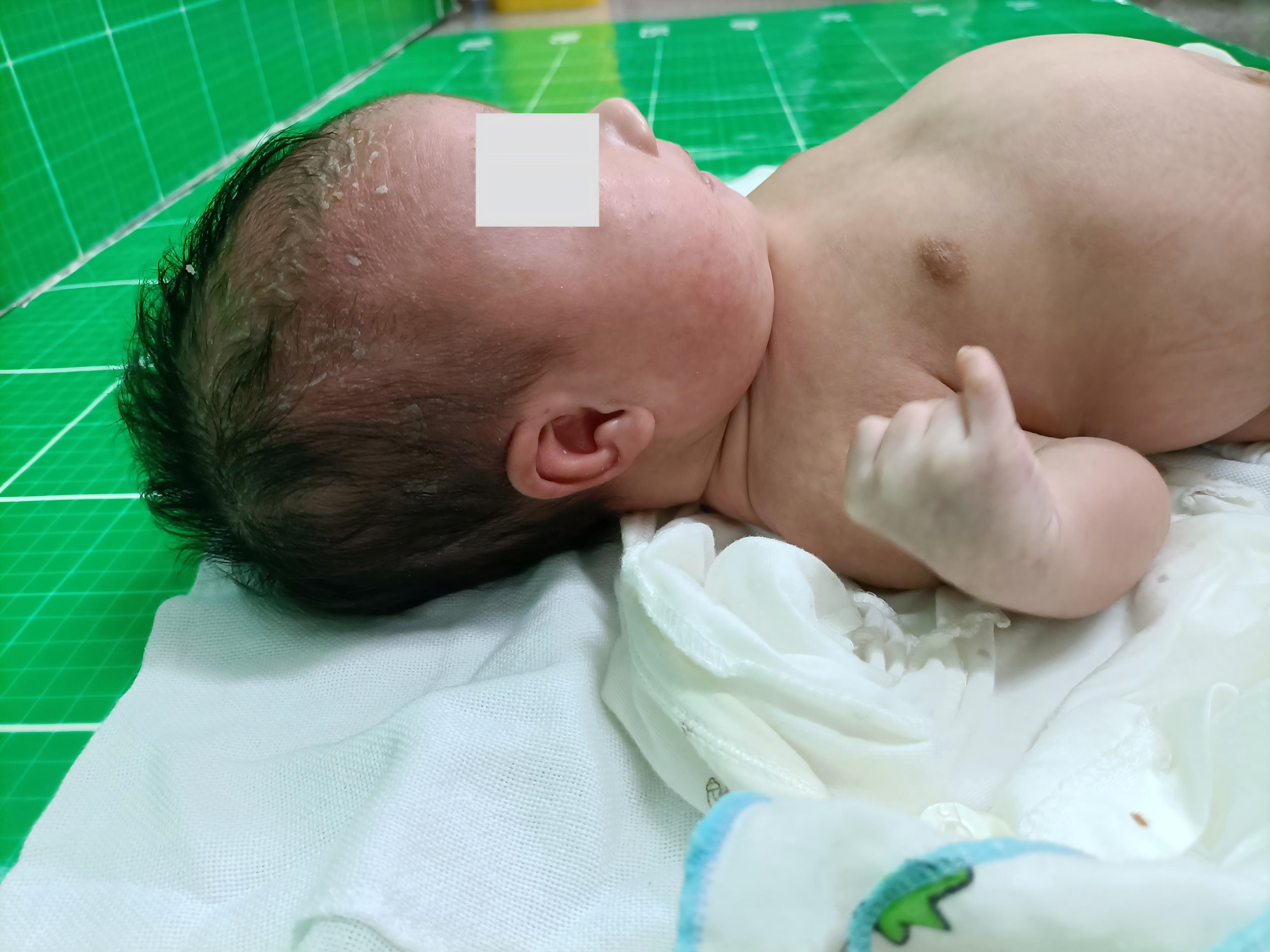

Supplement: Supporting Information 2 — Supporting Figure 1. Photograph of the 51-day-old boy with 49, XXXYY. [file 1368153.f2.jpg]

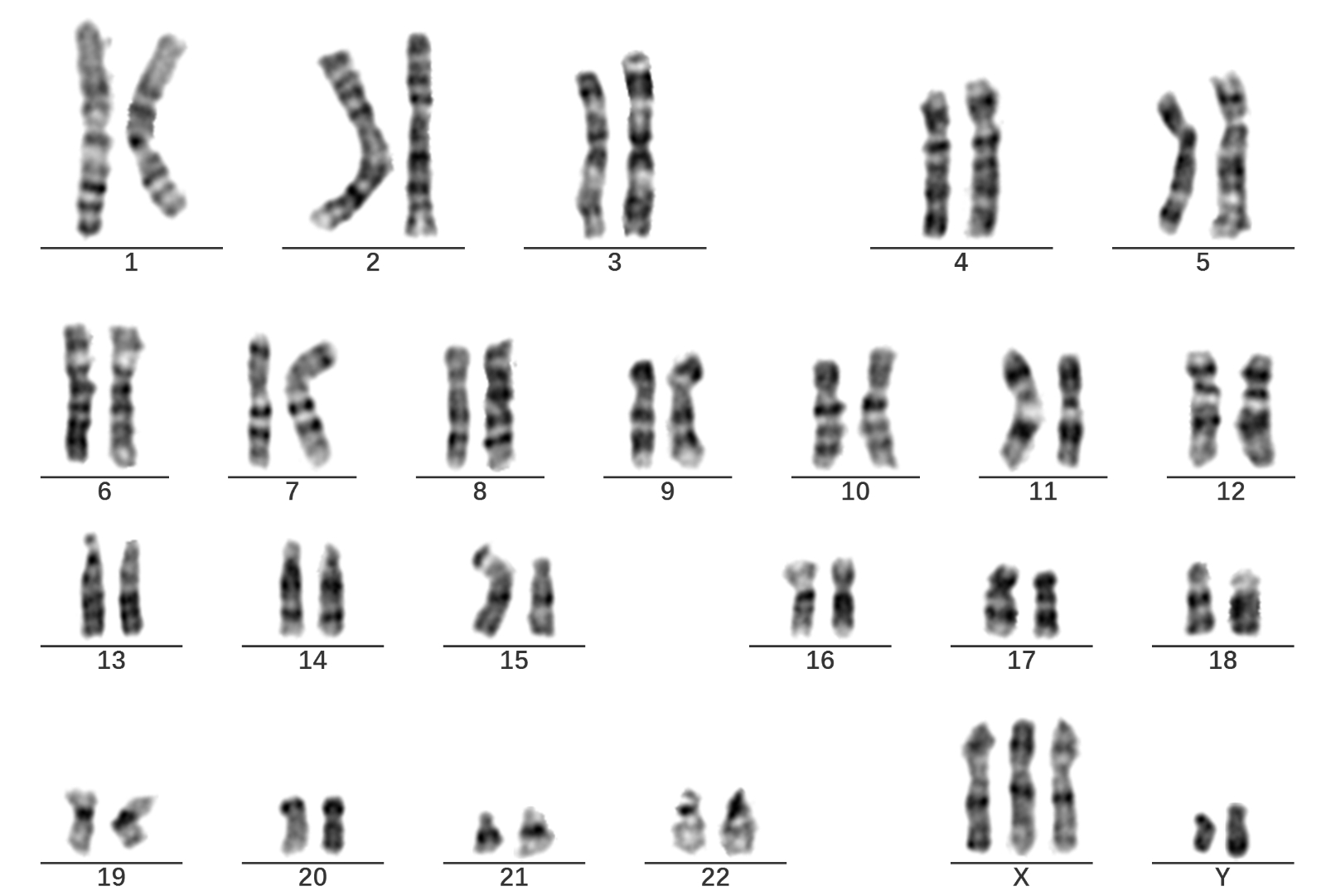

Supplement: Supporting Information 3 — Supporting Figure 2. Karyotype of the patient (49, XXXYY). [file 1368153.f3.JPG]

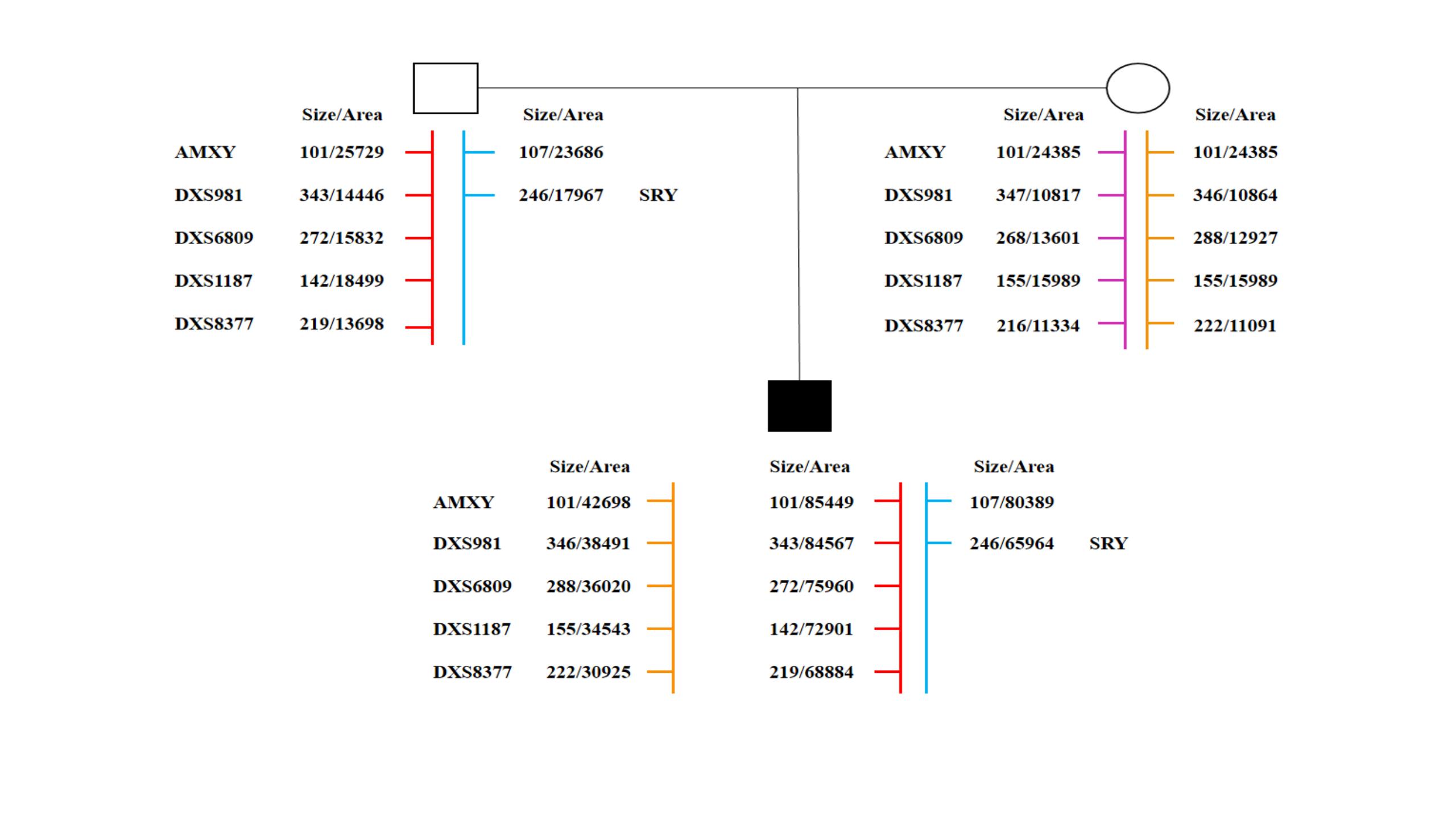

Supplement: Supporting Information 4 — Supporting Figure 3. Haploid typing-linkage analysis. [file 1368153.f4.jpg]
