# Supplementary material for: 49, XXXYY: Parental Origin, Occurrence, and Clinical Phenotypes
Source: Genet Res (Camb). 2025 Jul 21;2025:1368153. doi: 10.1155/genr/1368153 (PMC12316498; doi:10.1155/genr/1368153)
Supplement: Supporting Information 5 — Supporting Table 1. Results of STR. [file 1368153.f5.docx]

Supplemental Table 1. Results of STR

| **Locus/Gene** | **Location** | **Size of products (bp)** | **Size of products in father (bp)** | **Size of products in mather (bp)** | **Size of products in the patient (bp)** |
| --- | --- | --- | --- | --- | --- |
| D21S1433 | 21q21.3 | 140-190 | 154/158 | 158 | 154/158 |
| 21q11.2 | 21q11.2 | 170-220 | 187/197 | 184/193 | 193/197 |
| D21S1411 | 21q22.3 | 270-325 | 294/302 | 298/306 | 302/306 |
| D21S1414 | 21q21.1 | 315-370 | 343/357 | 343/355 | 343/355 |
| D21S1412 | 21q22.2 | 380-450 | 399/430 | 430/438 | 399/438 |
| D21S1445 | 21q22.11 | 470-530 | 495/508 | 488/512 | 488/508 |
| D18S1002 | 18q11.2 | 108-140 | 116 | 120 | 116/120 |
| D18S391 | 18p11.31 | 180-220 | 187/191 | 187/195 | 187 |
| D18S535 | 18q12.3 | 235-280 | 259/264 | 256 | 256/259 |
| D18S386 | 18q22.1 | 305-375 | 326/330 | 322/326 | 326/420 |
| D13S628 | 13q31.1 | 140-190 | 150 | 179/183 | 150/183 |
| D13S742 | 13q12.12 | 220-275 | 245/256 | 225/246 | 225/245 |
| D13S634 | 13q21.32 | 320-365 | 325/343 | 339 | 339/343 |
| D13S305 | 13q13.3 | 370-430 | 398/402 | 386/402 | 386/398 |
| DXS1187 | Xq26.2 | 130-170 | 142 | 155 | 142/155 |
| DXS8377 | Xq28 | 180-254 | 219 | 216/222 | 219/222 |
| DXS6809 | Xq21.33 | 255-300 | 272 | 268/288 | 272/288 |
| DXS981 | Xq13.1 | 310-370 | 343 | 346/347 | 343/346 |
| AMXY | Xp22.2 | 102 | 101 | 101 | 101 |
| AMXY | Yp11.2 | 108 | 107 | - | 107 |
| SRY | Yp11.2 | 248 | 246 | - | 246 |
